# Retraction: Acellularized spinal cord scaffolds incorporating bpV(pic)/PLGA microspheres promote axonal regeneration and functional recovery after spinal cord injury

**DOI:** 10.1039/d3ra90004e

**Published:** 2023-01-16

**Authors:** Jia Liu, Kai Li, Ke Huang, Chengliang Yang, Zhipeng Huang, Xingchang Zhao, Shiqiang Song, Taisen Pang, Jing Zhou, Yuhai Wang, Chong Wang, Yujin Tang

**Affiliations:** a Department of Orthopedics, Affiliated Hospital of Youjiang Medical University for Nationalities 18 Zhongshan II Road Baise Guangxi 533000 China tangyujin1967@163.com +86-0776-2833076; b Academy of Orthopedics, Guangdong Province, The Third Affiliated Hospital of Southern Medical University Guangzhou Guangdong 510000 China; c Department of Anatomy, Youjiang Medical College for Nationalities Baise Guangxi 533000 China; d Academy of Orthopedics, People’s Hospital of Ningxia Hui Autonomous Region Ningxia 502213 China; e School of Mechanical Engineering, Dongguan University of Technology No. 1 University Road, Songshan Lake Dongguan Guangdong 523808 P. R. China wangchong@dgut.edu.cn +86-1341-6885162

## Abstract

Retraction of ‘Acellularized spinal cord scaffolds incorporating bpV(pic)/PLGA microspheres promote axonal regeneration and functional recovery after spinal cord injury’ by Jia Liu *et al.*, *RSC Adv.*, 2020, **10**, 18677–18686, https://doi.org/10.1039/D0RA02661A.

The Royal Society of Chemistry hereby wholly retracts this *RSC Advances* article due to concerns with the reliability of the data.

Part of the image in Fig. 1g (Day 3, bpV(pic) panel) is a scaled, rotated and inverted version of the image in Fig. 3d (con, β-III-tubilin panel).

The authors also informed the editor that incorrect H&E staining images, labelled with bpV(pic)/PLGA-ASC, were used in Fig. 4c.

Given the significance of the concerns about the validity of the data in the article, the findings presented in this paper are not reliable.

The authors do not agree with the decision to retract the article.

Signed: Laura Fisher, Executive Editor, *RSC Advances*

Date: 22nd December 2022

## Supplementary Material

